# Rapid identification of bloodstream infection pathogens and drug resistance using Raman spectroscopy enhanced by convolutional neural networks

**DOI:** 10.3389/fmicb.2024.1428304

**Published:** 2024-07-15

**Authors:** Haiquan Kang, Ziling Wang, Jingfang Sun, Shuang Song, Lei Cheng, Yi Sun, Xingqi Pan, Changyu Wu, Ping Gong, Hongchun Li

**Affiliations:** ^1^Department of Clinical Laboratory, Affiliated Hospital of Xuzhou Medical University, Xuzhou, China; ^2^Medical Technology School, Xuzhou Medical University, Xuzhou, China; ^3^School of Medical Imaging, Xuzhou Medical University, Xuzhou, China

**Keywords:** bloodstream infection (BSI), Raman spectroscopy, convolutional neural network, pathogen, drug resistance

## Abstract

Bloodstream infections (BSIs) are a critical medical concern, characterized by elevated morbidity, mortality, extended hospital stays, substantial healthcare costs, and diagnostic challenges. The clinical outcomes for patients with BSI can be markedly improved through the prompt identification of the causative pathogens and their susceptibility to antibiotics and antimicrobial agents. Traditional BSI diagnosis via blood culture is often hindered by its lengthy incubation period and its limitations in detecting pathogenic bacteria and their resistance profiles. Surface-enhanced Raman scattering (SERS) has recently gained prominence as a rapid and effective technique for identifying pathogenic bacteria and assessing drug resistance. This method offers molecular fingerprinting with benefits such as rapidity, sensitivity, and non-destructiveness. The objective of this study was to integrate deep learning (DL) with SERS for the rapid identification of common pathogens and their resistance to drugs in BSIs. To assess the feasibility of combining DL with SERS for direct detection, erythrocyte lysis and differential centrifugation were employed to isolate bacteria from blood samples with positive blood cultures. A total of 12,046 and 11,968 SERS spectra were collected from the two methods using Raman spectroscopy and subsequently analyzed using DL algorithms. The findings reveal that convolutional neural networks (CNNs) exhibit considerable potential in identifying prevalent pathogens and their drug-resistant strains. The differential centrifugation technique outperformed erythrocyte lysis in bacterial isolation from blood, achieving a detection accuracy of 98.68% for pathogenic bacteria and an impressive 99.85% accuracy in identifying carbapenem-resistant *Klebsiella pneumoniae*. In summary, this research successfully developed an innovative approach by combining DL with SERS for the swift identification of pathogenic bacteria and their drug resistance in BSIs. This novel method holds the promise of significantly improving patient prognoses and optimizing healthcare efficiency. Its potential impact could be profound, potentially transforming the diagnostic and therapeutic landscape of BSIs.

## Introduction

Bloodstream infection (BSI) is a critical condition characterized by the invasion of pathogenic bacteria into the bloodstream, leading to their proliferation, toxin and metabolite secretion, and cytokine production. In severe cases, BSI can progress to systemic multiple organ dysfunction syndrome (MODS), shock, and disseminated intravascular coagulation, culminating in a high mortality rate. Common clinical manifestations include fever, chills, and hepatosplenomegaly (Zhang et al., 2020). Annually, nearly 2 million cases of BSI are reported in North America and Europe, with an estimated 250,000 resulting in death. In China alone, research in 2019 showed a link between bloodstream infections and more than half a million deaths (Zhang et al., 2023). This trend highlights the escalating impact of BSI as a significant public health issue globally (Goto and Al-Hasan, 2013). Timely administration of appropriate antibiotics is crucial for improving survival rates and patient outcomes. Studies, such as one by Liu et al., have shown that delays in treatment can lead to a 9% increase in mortality for each hour of delay (Liu V. X. et al., 2017). However, the current diagnostic gold standard for BSI, blood culture, is limited by its low sensitivity and the extended period required for results, typically ranging from 2 to 6 days, which can be detrimental in the context of urgent clinical needs.

To our current understanding, advanced molecular diagnostic techniques, including matrix-assisted laser desorption/ionization time-of-flight mass spectrometry (MALDI-ToF MS), polymerase chain reaction (PCR), and metagenomic next-generation sequencing (mNGS), have been instrumental in the rapid detection of pathogenic bacteria (Hu et al., 2021; Peri et al., 2022; Wang L. et al., 2023). MALDI-ToF MS has become a clinical mainstay for microbial identification, lauded for its ease of use, rapid analysis, cost-effectiveness, and its ability to identify a wide array of microorganisms, encompassing aerobic and anaerobic bacteria, fungi, and mycobacteria. Despite these advantages, MALDI-ToF MS encounters difficulties in distinguishing between closely related bacterial species and is less effective in identifying multiple microorganisms when they are growing in a mixture. The technique’s efficacy in pathogen identification is also dependent on the isolation of a pure culture. Additionally, the visible single colony after the blood culture has turned positive requires 18 to 24 h for growth, which often does not meet the demand for a rapid diagnosis in clinical settings (Peri et al., 2022).

The polymerase chain reaction (PCR)-based diagnostic system is renowned for its exceptional detection rate, which is a notable 13 times higher than that of traditional blood culture methods. This technique’s resilience to the presence of antibiotics renders it highly effective in identifying complex microbial infections, a challenge often encountered with blood culture techniques (Suberviola et al., 2016). Despite these strengths, PCR-based detection in blood samples confronts several hurdles. The low pathogen load in blood samples and the abundance of background DNA, along with PCR-inhibitory substances, can diminish the sensitivity of molecular detection methods. Overcoming these obstacles is essential for bolstering the reliability and precision of PCR-based diagnostics in clinical practice. Metagenomic next-generation sequencing (mNGS) has significantly expanded the horizons of pathogen detection. Capable of identifying all genomic material present, mNGS is adept at uncovering a wide spectrum of pathogens, with a detection rate that surpasses that of conventional methods. However, the mNGS process is complex, necessitates expensive reagents, and requires a sophisticated laboratory environment, which may hinder its accessibility and speed of execution (Wang L. et al., 2023).

Traditional antibiotic drug sensitivity testing methods, including micro broth dilution, disc diffusion, gradient diffusion, and various commercial automated systems, are often predicated on the availability of pure cultures. This requirement can extend the diagnostic process, potentially delaying the critical detection and treatment of patients in clinical settings. Despite the advancements in modern assays such as mass spectrometry (MS), PCR, and metagenomic next-generation sequencing (mNGS), which have enhanced diagnostic timelines and sensitivity, these methods are not without their challenges. They often contend with issues related to automation, cost-effectiveness, and operational simplicity. Given these constraints, there is an acute need for a diagnostic technique for bloodstream infections (BSIs) that is rapid, user-friendly, and highly efficient. The development of such a method could be revolutionary, significantly improving the speed and effectiveness of patient care in response to BSIs.

Surface-enhanced Raman scattering (SERS), a method based on the principles of Raman scattering, has emerged as a highly promising tool for the identification of pathogenic microorganisms. SERS is distinguished by its capacity to furnish intricate molecular structural information and to significantly enhance Raman scattering signals, thereby improving detection sensitivity. The technique is advantageous for several reasons: it is user-friendly, facilitates rapid analysis, is cost-effective, allows for non-destructive sample examination, and is resilient against interference from moisture (Xia et al., 2022). A pivotal study conducted by Zhou et al. (2014) achieved a significant advancement in SERS, increasing its sensitivity by 30-fold. This was made possible through the *in situ* synthesis of silver nanoparticles (AgNPs) on bacterial cell walls, effectively differentiating between various strains of *Escherichia coli* and *Staphylococcus epidermidis*. Further progress was made by Dekter et al. (2017), who demonstrated the effectiveness of Raman spectroscopy combined with a clustering algorithm for analyzing drug sensitivity profiles. Their results were found to be in close alignment with those from VITEK^®^ 2 systems and traditional broth dilution methods. Despite these significant strides, traditional SERS spectroscopy encounters challenges in differentiating closely related bacterial species, often due to the complex and time-consuming nature of the preprocessing steps required. To fully leverage the potential of SERS in microbial identification and profiling of drug sensitivity, there is an urgent requirement for the development and adoption of a classification algorithm that is both efficient and highly accurate.

Machine learning techniques, particularly support vector machines (SVMs), have proven effective in Raman spectroscopy for identifying specific pathogens, including *Mycobacterium tuberculosis* (Stöckel et al., 2017). However, these traditional approaches often necessitate extensive preprocessing of spectral data, which can be time-consuming and complex. Deep learning (DL) presents a compelling alternative, with the capacity to automatically discern characteristic spectra from Raman data. Prior research has shown that DL can achieve high performance in classifying not only bacteria to the genus level but to the species level (Wang et al., 2022).

The direct detection of microorganisms from blood samples is a challenging task due to the low concentration of pathogens and the intricate nature of the blood matrix. To bolster the efficacy and precision of diagnostic methods, this study has assessed two separation techniques: differential centrifugation and erythrocyte lysis. The investigation centers on their potential to enhance the direct isolation of bacteria from positive blood culture samples, which could lead to more accurate identification and analysis of pathogens in bloodstream infections. Given that many current detection methods depend on the time-consuming process of obtaining a pure culture, this study also explores the integration of convolutional neural networks (CNNs) with SERS technology. The objective is to directly identify common pathogens and their drug resistance profiles in bloodstream infections, thereby providing a diagnostic approach that is both more efficient and reliable.

## Materials and methods

### Chemical and biological materials

Positive blood culture bottles were sourced from the microbiology laboratory of the Affiliated Hospital of Xuzhou Medical University, and collected over the period from June to September 2023. The study applied specific inclusion and exclusion criteria to ensure the relevance and accuracy of the samples analyzed. The inclusion criteria encompassed the following: (1) the specimen type was blood; (2) common clinical pathogens, namely *Escherichia coli* (*E. coli*), *Klebsiella pneumoniae* (*K. pneumoniae*), *Acinetobacter baumannii* (*A. baumannii*), *Enterococcus faecium* (*E. faecium*), *Enterococcus faecalis* (*E. faecalis*), *Staphylococcus aureus* (*S. aureus*), and *Pseudomonas aeruginosa* (*P. aeruginosa*), were identified using a MALDI-TOF MS mass spectrometer following inoculation and culture.

The exclusion criteria were designed to maintain the study’s focus and ensure the validity of the results, and included: (1) blood cultures derived from different regions of the same patient with Only one blood culture per patient included in the study; (2) samples with mixed infections involving multiple bacterial strains were excluded. Upon a positive result from the blood incubator (bioMérieux, France), blood samples underwent smear gram staining and were examined microscopically. Subsequently, the samples were inoculated onto blood agar plates and incubated at 35°C until the formation of single colonies. The bacterial strains were then definitively identified using MALDI-TOF MS mass spectrometry (Bruker, Germany). Antibiotic sensitivity profiles were determined with the VITEK-2 Compact Automated Microbial Identification and Drug Sensitivity Analyzer (bioMérieux, France). The findings from routine pathogen identification and drug sensitivity testing served as the benchmark for reference in this study.

### Ethical considerations

The study was conducted with the approval of the Ethics Committee of Xuzhou Medical University Hospital, ensuring that all procedures were in compliance with ethical standards.

### Bacterial sample preparation

**Figure 1:**
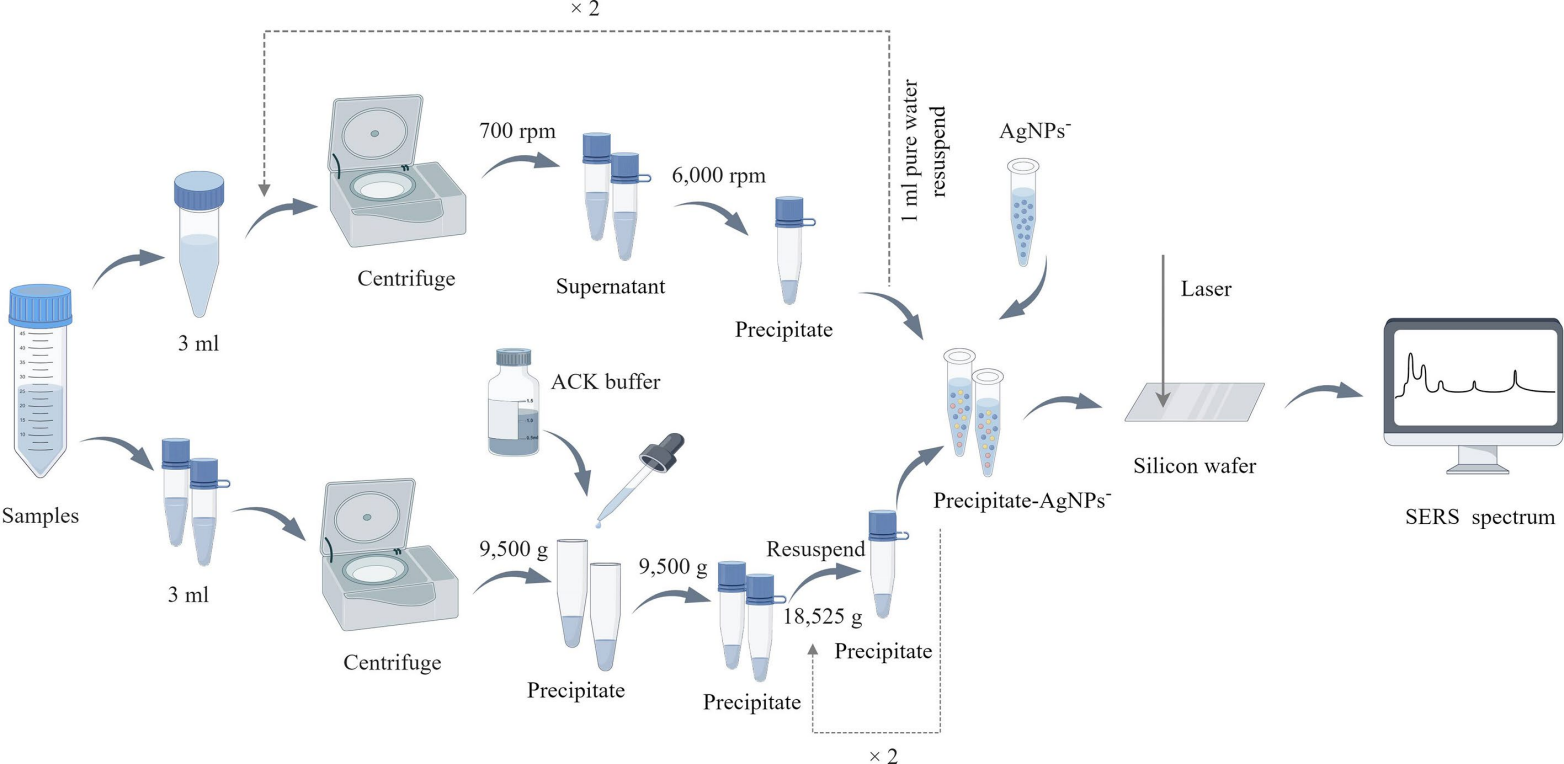
Bacterial sample preparation flowchart. In the upper part of the figure is the processing flow of the differential centrifugation method, and the lower part is the erythrocyte lysis method, this figure is drawn by Figdraw (www.figdraw.com).

Two distinct methods were employed to extract bacterial particles from positive blood culture bottles for SERS analysis: differential centrifugation and erythrocyte lysis (Figure 1). Both methods aimed to isolate bacterial particles for SERS analysis while minimizing the presence of blood components that could interfere with the detection of bacterial signals. (1) The differential centrifugation method: the blood sample (3 mL) was drawn from the positive blood culture bottle via a sterile syringe. The sample was centrifuged at 700 rpm for 10 min. The supernatant was collected and again centrifuged at 6,000 rpm for 10 min. After discarding the supernatant, the pellet was resuspended in 1 mL of deionized water and centrifuged at 700 rpm for 10 min, followed by another centrifugation at 6,000 rpm for 10 min, the pellet was precipitated and stored. (2) The erythrocyte lysis method: the blood sample (3 mL) was drawn from the positive blood culture bottle via a sterile syringe. The sample was centrifuged at 9,500 g for 5 min. After discarding the supernatant, 3 mL of ACK erythrocyte lysate (Han et al., 2020) was added, mixed, and incubated for 10 min. After incubation, the sample was centrifuged at 9,500 g for 5 min, the supernatant was discarded and the pallet was resuspened into 2 mL of deionized water. The sample was again centrifuged at 18,525 g for 2 min and the pellet was stored.

### Preparation of negatively charged nano-silver-enhanced substrate

A solution was prepared by mixing 200 mL of ultrapure water with 33.72 mg of AgNO_3_ in a sterile triangular flask that was heated to boiling while mixing with a magnetic stirrer. Next, 8 mL of a sodium citrate solution (1 wt%) was added and stirred at 650 r/min for 40 min with continued heating. Once the heating was halted, the stirring was maintained as the solution gradually cooled to the ambient temperature. The solution was adjusted to 200 mL, mixed properly, and then stored at 4°C in the dark for later use. Take 1 mL of the above solution in a 1.5 mL EP tube, centrifuge at 7,000 rpm for 7 min, discard the supernatant, resuspend it with 100 μL of ultrapure water, mix well, and set aside.

### Raman spectroscopic measurements

To ensure the intensity of the SERS signal, we prepared higher concentrations of bacterial suspensions. The particles obtained by both methods were mixed with 20 μL of deionized water and 20 μL of uniformly dispersed negatively charged silver nanoparticles (NPs) (the bacterial concentration exceeded 10^9^cfu/ml). The mixture was then incubated for 15 min. This mixture (7 mL) was poured onto a silicon wafer, allowed to dry in a biosafety cabinet, and promptly analyzed using a portable Raman spectrometer (B&W TEK, i-Raman Plus BWS 465-785H, America). For each clinical sample, 50 to 100 Raman spectra were obtained by spectral detection. The measurement parameters were set as follows: excitation light source of 785 nm, excitation power of 20 mW, integration time of 5 s, and detection spectral range of 500 to 1,800 cm^−1^. Before spectrum collection, it was necessary to measure the spectra at 520 cm^−1^ using a blank silicon wafer, while simultaneously subtracting the dark current during the integration time. The spectra were collected using the BWSpec software, and 12,046 and 11,968 spectra were collected by differential centrifugation and erythrocyte lysis, respectively.

### Averaged SERS spectra and characteristic peaks

The surface composition of bacteria is intricate and comprises various biomolecules, including proteins, lipids, polysaccharides, and other biochemical components. Spectral features can potentially be employed to identify bacterial species, while distinct Raman spectral signals may correspond to various molecular structures and represent the biochemical properties of biomolecules related to the bacteria (Zhao et al., 2023). The average SERS spectra and standard deviation of each bacterium were calculated and visualized using Origin 2023. To identify the distinctive peaks, the mean SERS spectra were imported into LabSpec 5 software (HORIBA Scientific, Japan). The spectra were further smoothed, baseline corrected, and normalized. The GaussLoren function was employed to detect the characteristic peaks in each mean SERS spectrum. The parameters for searching the characteristic peaks were set as level (%) = 10, size (pnt) = 15, and iteration = 5. Finally, dot plots were used to illustrate the distribution of the distinctive peaks present in each bacterial spectrum and elucidate their biological significance based on the reference.

### Construction of CNN model

**Figure 2:**
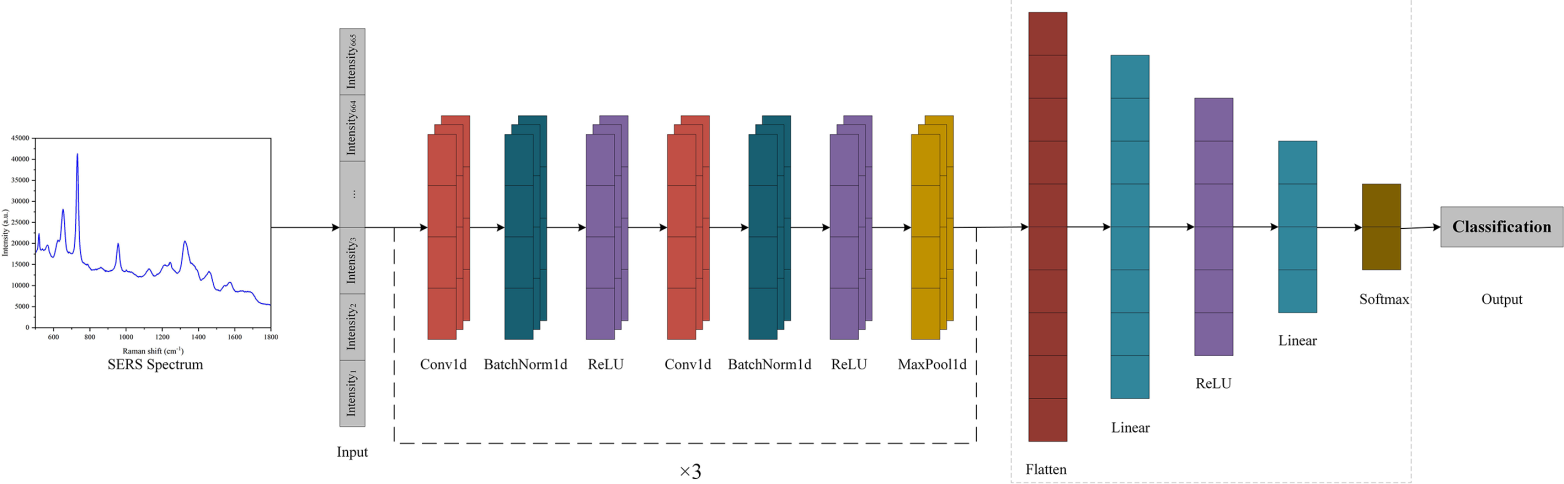
Schematic flow of CNN model for processing SERS spectra.

In this study, a SERS-CNN model was constructed to automatically classify and estimate the SERS spectra of seven pathogenic and antibiotic-resistant strains to implement the SERS method for the immediate identification of common pathogens and their drug resistance in BSIs. To enhance the application of computer recognition and DL algorithms, all pathogenic and antibiotic-resistant bacteria were detected with specific labels. Further, the LabelEncoder function was used to convert the pathogenic labels into discrete numerical variables. All the SERS spectral data were divided into training and test sets with the 5-fold cross-validation in the ratio of 8:2. The PyTorch DL framework was employed to construct the CNN model (Figure 2), which mainly consists of 6 convolution layers, 6 batchnorm layers, 3 max-pooling layers, 1 flatten layer and 2 fully connected layers (linear layers). The convolutional layers have a convolutional kernel size of 3 × 1, and the pooling layers have a sliding window size of 3 × 1. To expedite the training speed, improve the capacity of network expression, and minimize overfitting, a batch normalization layer, and a ReLU activation function were incorporated after each convolutional layer. The addition of a pooling layer enhances the ability of the model to learn local features and global features. Next, the flat layer transforms the multidimensional feature representation into a unidimensional form to enter the output of the pooling layer into the fully connected layer. Finally, the fully linked layer converts these attributes into the corresponding output categories. The model employed cross entropy as the loss function and used the SGD optimizer.

### CNN model evaluations

To assess the classification and prediction potential of CNN on SERS spectra, this study selected three traditional machine learning algorithms to compare with CNN, namely, random forest (RF), support vector machine (SVM), and Adaboost. These algorithms were implemented using Scikit-learn with the default parameter configurations. Moreover, choosing appropriate metrics is crucial to evaluate the effectiveness of machine learning. In the current study, accuracy (ACC), precision (Pre), recall (Recall), and F1 scores were selected to score several algorithms. Accuracy denotes the percentage of accurate outcome predictions, Precision signifies the proportion of samples expected to be in the positive category that indeed belong in the positive category, and Recall represents the proportion of predicted positive categories that are positive categories. Subsequently, the performance of multiple machine learning models was evaluated using the method of five-fold cross-validation. The evaluation process was as follows: first, the sequence of data was randomly shuffled. Then, the data was evenly divided into five subsets. For each iteration, a different subset was selected as the test set to assess the model’s performance, while the remaining subsets were used as the training set for model training. This process was repeated five times, and the final evaluation result was obtained by averaging the results from the five test sets.

## Results

### SERS spectra of 7 pathogenic bacteria

**Figure 3:**
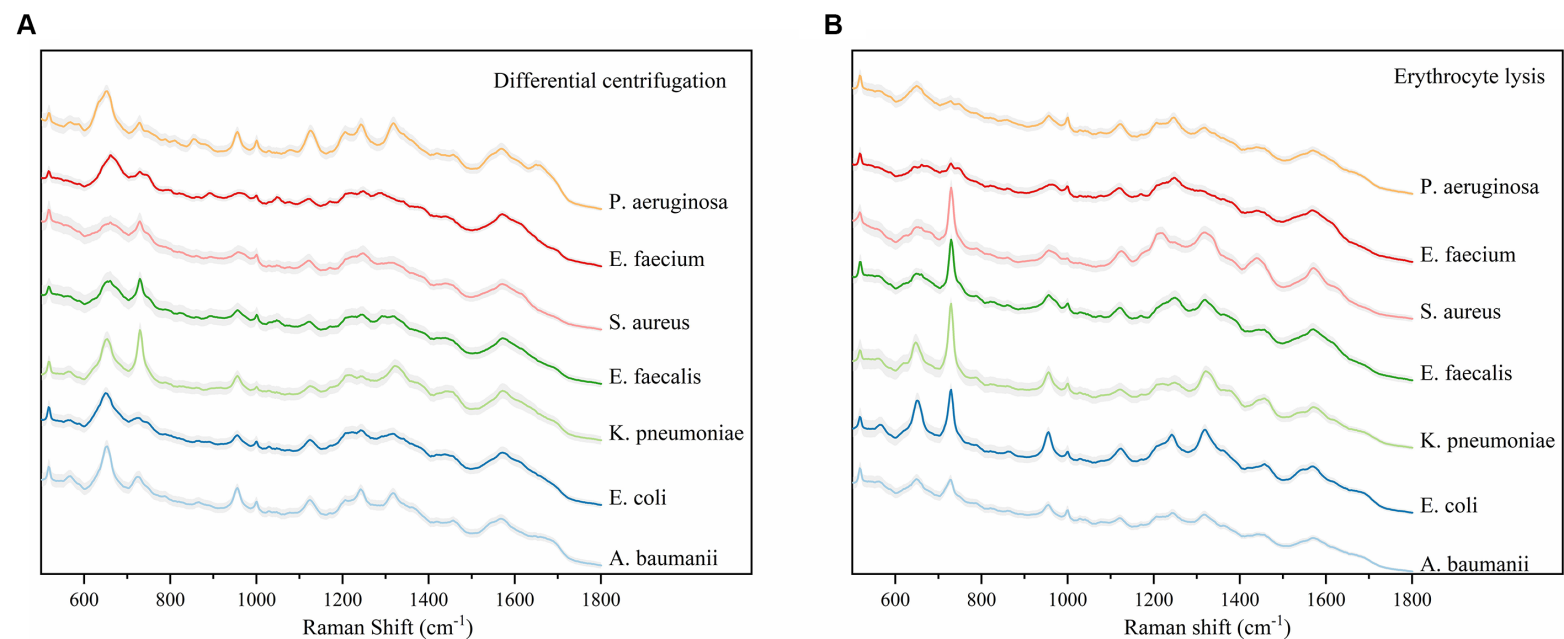
Average Raman spectra of the two methods and seven bacteria, the shaded area represents 20% of the standard deviation. **(A)** Average SERS spectra for the differential centrifugation method. **(B)** Average SERS spectra of the erythrocyte lysis method. Horizontal coordinates indicate Raman shifts.

**Figure 4:**
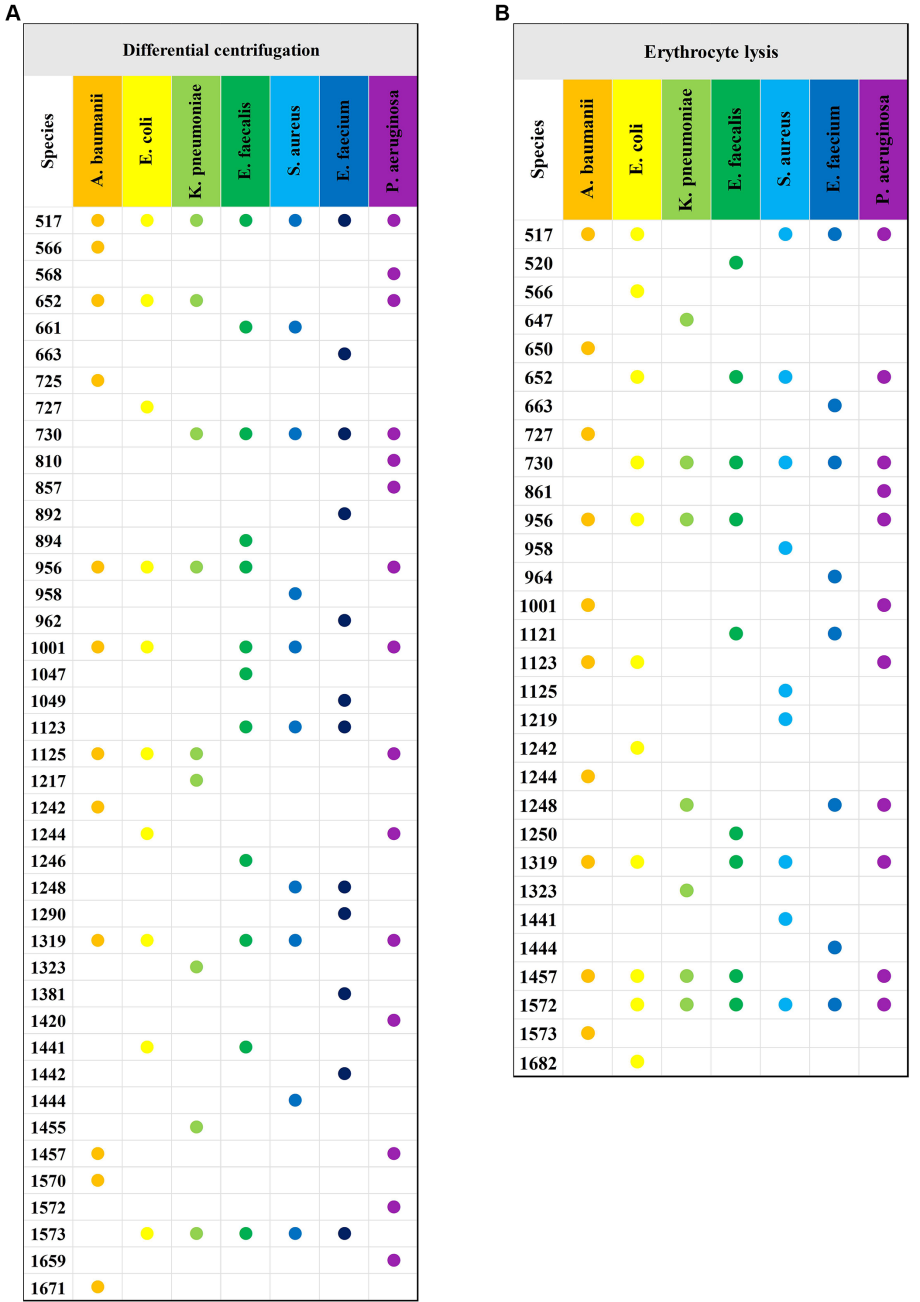
Characteristic peaks of the two methods and seven bacteria. **(A)** Characteristic peaks of the differential centrifugation method. **(B)** Characteristic peaks of the erythrocyte lysis method. The horizontal axis indicates the different species of bacteria, the vertical axis indicates the Raman displacements where the characteristic peaks are located, and the Raman displacements where the characteristic peaks of different species of bacteria are located are marked with solid dots of different colors.

The study analyzed a total of 130 blood culture bottles, which tested positive for the presence of bacteria, leading to the identification of seven distinct bacterial species. The breakdown of the bacterial isolates is as follows: *E. coli* with 43 isolates (*N*1 = 4,006 spectra from differential centrifugation, *N*2 = 3,953 spectra from erythrocyte lysis), *K. pneumoniae* with 28 isolates (*N*1 = 2,704, *N*2 = 2,650), *A. baumannii* with 16 isolates (*N*1 = 1,498, *N*2 = 1,342), *E. faecium* with 10 isolates (*N*1 = 908, *N*2 = 969), *E. faecalis* with 7 isolates (*N*1 = 605, *N*2 = 665), *S. aureus* with 13 isolates (*N*1 = 1,110, *N*2 = 1,224), and *P. aeruginosa* with 13 isolates (*N*1 = 1,215, *N*2 = 1,165) (Table 1). Here, “*n*” represents the number of blood culture vials, “*N*1” denotes the number of spectra acquired by differential centrifugation, and “*N*2” signifies the number of spectra acquired by erythrocyte lysis. Figure 3 presents the mean SERS spectra for the two extraction methods across the seven bacterial samples, with the shaded area representing the standard deviation (SD) of the spectra. Figure 3A illustrates the mean SERS spectra derived from the differential centrifugation method, whereas Figure 3B represents the mean SERS spectra from the erythrocyte lysis method. Additionally, Figure 4 provides dot plots that highlight the characteristic peaks in the average spectra of each bacterial species for both methods. Figure 4A corresponds to the dot plots from the differential centrifugation method, and Figure 4B corresponds to those from the erythrocyte lysis method. Table 2 summarizes the spectral band distribution of the main Raman characteristic peaks associated with the seven pathogenic bacteria, as documented in the literature.

**Table 1:** Information on the dataset of 7 bacterial species.

Species	Clinical samples (*n*)	Differential centrifugation (*N*1)			Erythrocyte lysis (*N*2)		
		Training data	Testing data	In total	Training data	Testing data	In total
*A. baumanii*	16	1,198	300	1,498	1,074	268	1,342
*E. coli*	43	3,205	801	4,006	3,162	791	3,953
*K. pneumoniae*	28	2,163	541	2,704	2,120	530	2,650
*E. faecalis*	7	484	121	605	532	133	665
*S. aureus*	13	888	222	1,110	979	245	1,224
*E. faecium*	10	726	182	908	775	194	969
*P. aeruginosa*	13	972	243	1,215	932	233	1,165
In total	130	9,636	2,410	12,046	9,574	2,394	11,968
CRKP	8	624	156	780	601	150	751
CSKP	20	1,539	385	1,924	1,519	380	1,899

*n* is the number of blood culture vials collected, *N*1 is the number of spectra obtained by differential centrifugation, *N*2 is the number of spectra obtained by erythrocyte lysis.

**Table 2:** Attribution of major Raman characteristic peaks of pathogens.

Raman shift (cm^−1^)	Band assignment	References
517/520	S–S stretch	Kahraman et al. (2008)
566/568	Carbohydrates	Kahraman et al. (2008) and Zhou et al. (2014)
647/650/652/661/663	δ(COO–) guanine	Walter et al. (2011), Zhou et al. (2014), and Chen et al. (2019)
725/727/730	Adenine, glycosidic ring mode	Walter et al. (2011) and Zhou et al. (2014)
810	υ(CN) tyrosin, porine, valin	Zhou et al. (2014)
857	Tyrosine	Liu Y. et al. (2017), Wang et al. (2022)
861	υ(C–C) skeletal proteins	Chen et al. (2019)
892/894	Phosphodiester backbone, deoxyribose	Chen et al. (2015)
956/958	υ(CN), protein	Walter et al. (2011) and Zhou et al. (2014)
962/964	N–C stretching	Chen et al. (2015)
1,001	“Breathing” in aromatic rings	Chen et al. (2019)
1047/1049	Carbohydrates	Walter et al. (2011) and Wang et al. (2022)
1121/1123/1125	υ(C–C) skeletal of acyl backbone	Wang et al. (2022)
1217/1219/1242/1244/1246/1248	Amide III or adenine, polyadenine and DNA	Ivleva et al. (2010) and Chen et al. (2019)
1250/1290	Amide III	Knauer et al. (2010) and Zhou et al. (2014)
1319/1323	υ(NH_2_) adenine, polyadenine, DNA	Chen et al. (2019)
1381/1420	υ(COO–) symmetric	Knauer et al. (2010) and Zhou et al. (2014)
1441/1442/1444/1455/1457	δ(CH_2_) saturated lipids	Knauer et al. (2010) and Zhou et al. (2014)
1570/1572/1573	Amide II, υ(CN), γ(NH)	Knauer et al. (2010) and Zhou et al. (2014)
1659/1671/1682	Amide I	Knauer et al. (2010) and Zhou et al. (2014)

### Raman spectra of drug-resistant bacteria

**Figure 5:**
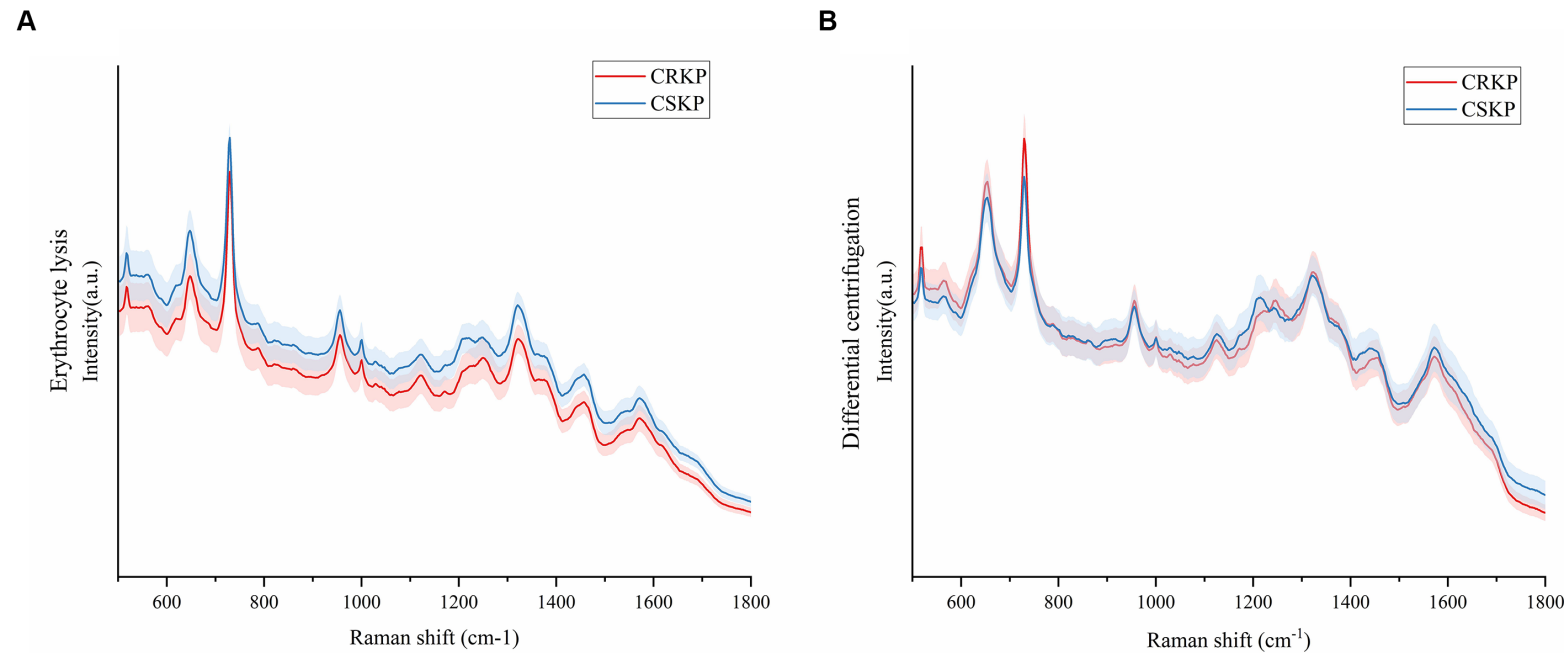
Mean Raman spectra of CRKP and CSKP, shaded areas represent 20% of the standard deviation. **(A)** Average SERS spectra from the erythrocyte lysis method. **(B)** Average SERS spectra of the differential centrifugation method. Horizontal coordinates represent Raman shifts and vertical coordinates represent intensities.

This study aimed to assess the capability of SERS for the direct detection of antibiotic-resistant bacteria in bloodstream infections (BSIs). For this purpose, a total of 8 cases of carbapenem-resistant *Klebsiella pneumoniae* (CRKP) and 20 cases of carbapenem-sensitive *Klebsiella pneumoniae* (CSKP) were selected from the blood culture specimens. These strains were identified using standard microbiological procedures. The SERS spectra for both CRKP and CSKP strains, obtained through differential centrifugation and erythrocyte lysis methods, were computed to derive average spectra. These average spectra are depicted in Figure 5, which presents a comparative analysis of the spectral features associated with resistant and sensitive strains of *K. pneumoniae*.

### Machine learning analysis of SERS spectra

In this study, the analysis of SERS spectral data was conducted using four distinct machine learning methods: convolutional neural network (CNN), random forest (RF), support vector machine (SVM), and Adaboost. The performance of these algorithms was rigorously evaluated based on their accuracy (ACC), precision (Pre), recall, and F1 scores. The results, as detailed in Tables 3–6, provide a comprehensive overview of the algorithmic analyses. Table 3 highlights that the CNN exhibited the most exceptional performance in classifying the SERS spectra of seven bacterial species obtained through differential centrifugation. The CNN achieved an accuracy of 98.68%, a precision of 98.71%, a recall of 98.68%, and an F1 score of 98.67%. Table 4 shows that the CNN model also outperformed others in classifying the SERS spectra of the same seven bacterial species when obtained through erythrocyte lysis, with an accuracy of 95.75%, a precision of 94.92%, a recall of 95.75%, and an F1 score of 95.14%. A comparison of the results presented in Tables 3, 4 reveals that the CNN consistently demonstrated superior classification performance across the board, suggesting its efficacy in prognostication for pathogenic bacteria. Moreover, the data indicate that differential centrifugation is a more effective technique for the separation of bacteria from positive blood culture bottles when compared to erythrocyte lysis.

**Table 3:** Comparison of classification and prediction ability of four machine learning methods on SERS spectra of seven bacteria obtained by differential centrifugation method.

Algorithm	ACC	Pre	Recall	F1
CNN	98.68%	98.71%	98.68%	98.67%
RF	95.48%	95.56%	95.48%	95.46%
SVM	82.34%	83.97%	82.34%	81.66%
Adaboost	52.52%	53.00%	52.52%	50.68%

**Table 4:** Comparison of the classification and prediction ability of four machine learning methods on SERS spectra of seven bacteria obtained by erythrocyte lysis method.

Algorithm	ACC	Pre	Recall	F1
CNN	95.75%	94.92%	95.75%	95.14%
RF	94.72%	94.76%	94.72%	94.71%
SVM	77.84%	78.90%	77.84%	77.09%
Adaboost	51.86%	53.05%	51.86%	50.98%

Furthermore, the CNN model’s classification performance was superior to that of RF, SVM, and Adaboost in analyzing the SERS spectra of pathogenic bacteria. Tables 5, 6 present the outcomes of the algorithms employed to identify drug-resistant bacteria. The CNN model showed high accuracy in classifying both CRKP and CSKP from SERS spectra, regardless of the separation method used. Notably, the SERS spectra obtained through differential centrifugation achieved a slightly higher accuracy of 99.85% compared to the 99.13% accuracy from erythrocyte lysis SERS spectra.

**Table 5:** Comparison of classification and prediction abilities of four machine learning methods for SERS spectra of drug-resistant bacteria obtained by differential centrifugation method.

Algorithm	ACC	Pre	Recall	F1
CNN	99.85%	99.86%	99.85%	99.85%
RF	99.00%	99.00%	99.00%	99.00%
SVM	90.79%	91.17%	90.79%	90.34%
Adaboost	98.52%	98.52%	98.52%	98.52%

**Table 6:** Comparison of classification and prediction ability of SERS spectra of drug-resistant bacteria obtained by erythrocyte lysis of four machine learning methods.

Algorithm	ACC	Pre	Recall	F1
CNN	99.13%	99.14%	99.13%	99.13%
RF	97.89%	97.90%	97.89%	97.87%
SVM	81.62%	83.84%	81.62%	78.72%
Adaboost	97.17%	97.17%	97.17%	97.16%

**Figure 6:**
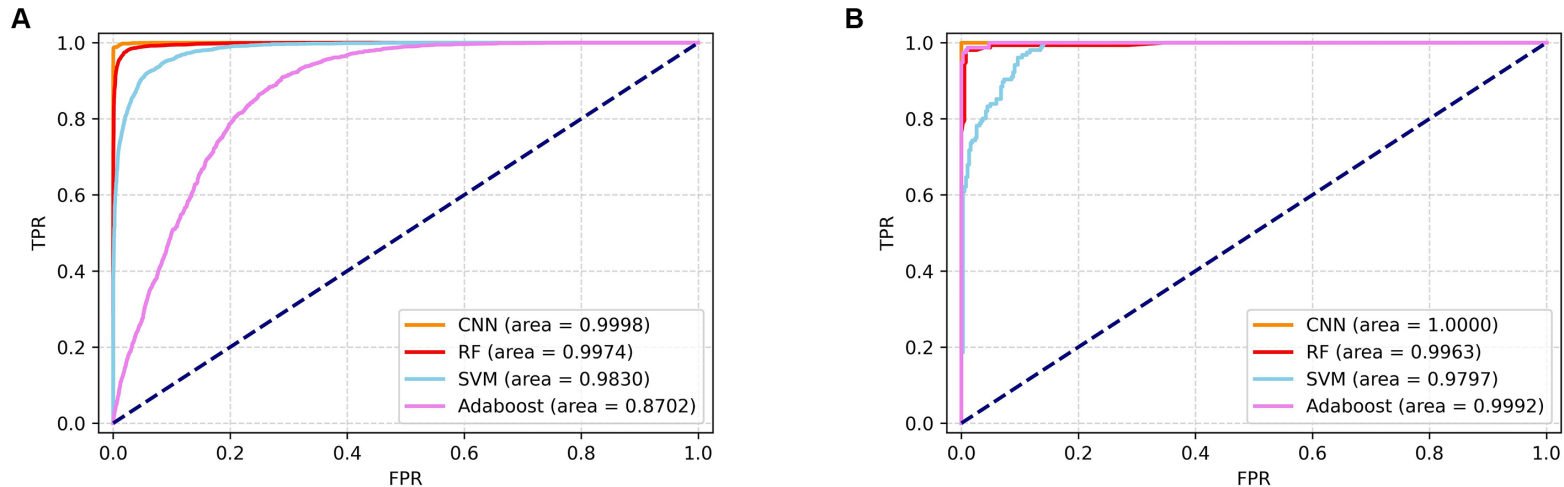
ROC zone curves of SERS spectrograms obtained by differential centrifugation classified by four algorithms. **(A)** ROC curves of four algorithms for the identification of seven pathogenic bacteria. **(B)** ROC curves of four algorithms for the detection of CRKP and CSKP.

The tables present the results of the differential centrifugation method for SERS spectral classification, which demonstrates superior performance. Furthermore, to assess the predictive capability of the classification model, we generated subject operating characteristic (ROC) curves for all the machine learning approaches (Figure 6). The ROC curves are used to assess the performance of the classifier. The area under the ROC curves is denoted as the AUC; a higher AUC value indicates superior model classification performance. Further, the ROC curve point in the closest distance to the upper-left corner demonstrates the highest sensitivity and specificity. In the upper left corner, a higher AUC value indicates better classification performance of the model. The point on the ROC curve that was closest to the upper left corner showed the highest sensitivity and specificity, as depicted in the figure. Based on the SERS spectrum classification obtained via differential centrifugation, it is evident that the CNN algorithm shows the highest performance in classification and prediction ability, as indicated by its ROC curve being closest to the upper right corner among the four algorithms.

**Figure 7:**
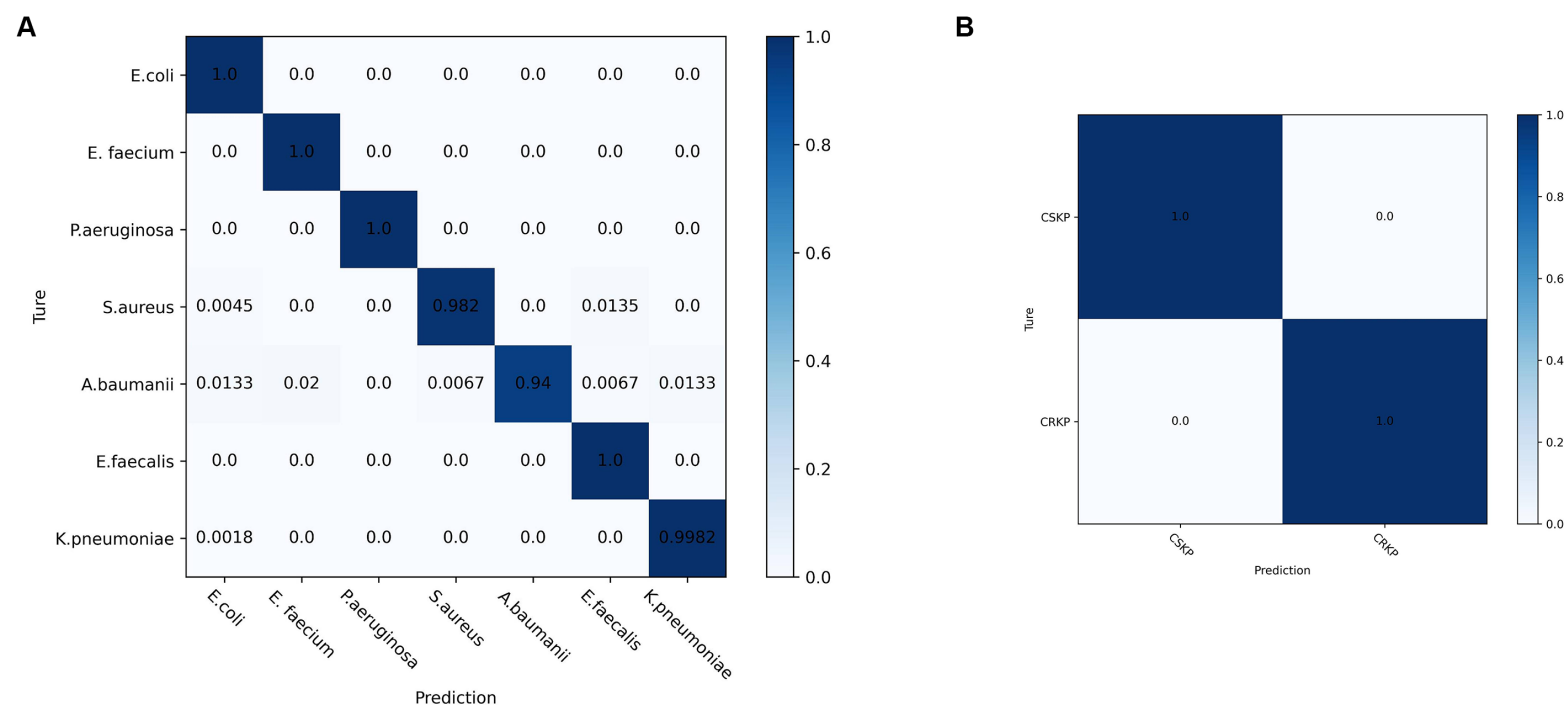
Confusion matrix of SERS spectra of pathogenic and drug-resistant bacteria obtained by CNN model classification differential centrifugation. **(A)** Confusion matrix of SERS spectra of seven pathogenic bacteria detected by CNN. **(B)** Confusion matrix of SERS spectra of CRKP and CSKP detected by CNN.

Lastly, this study selected the CNN model that yielded the most accurate classification outcomes and used the spectra acquired through differential centrifugation, which proved to be the most effective method for isolating bacteria, to generate the confusion matrix (Figure 7). The matrix’s vertical axis corresponds to the bacterial species and drug resistance detected by the reference method, while the horizontal axis corresponds to the bacterial identifications and drug resistance by the CNN algorithm. As illustrated in Figure 7A, the outcomes of CNN identification for *E. coli*, *E. faecium*, *P. aeruginosa*, and *E. faecalis* were consistent with those identified by the reference method, as determined by differential centrifugation of the SERS spectra. The accuracy of *S. aureus* identified by CNN was 98.2%, with 0.45% of SERS spectra being mistaken for *E. coli*. The CNN predicted *A. baumanii* with an accuracy of 94%, and the remaining 1.33% of the SERS spectra were misidentified as *E. coli*, 2% as *E. faecium*, 0.67% as *S. aureus*, 0.67% as *E. faecalis*, 1.33% as *S. aureus*, and 1.33% as *E. faecalis*, 1.33% were misclassified as *K. pneumoniae*. The CNN predicted *K. pneumoniae* with 99.82% accuracy and the remaining 0.18% were misclassified as *E. coli*. Figure 7B shows the confusion matrix of CRKP and CSKP obtained by CNN detection of differential centrifugation. The CNN accurately and precisely identifies CRKP and CSKP, with findings that align perfectly with the reference method.

## Discussion

SERS is an analytical technique renowned for its rapidity, non-destructiveness, and exceptional sensitivity, making it an ideal candidate for early clinical diagnosis. SERS offers several distinct advantages for the detection of biological samples. It provides detailed molecular structural information, which is crucial for pathogen identification. The technique also eliminates the need for extensive sample pretreatment, saving valuable time. Raman spectroscopy improves early identification of Candida and contributes to early optimal antifungal therapy (Chouthai et al., 2015). SERS analysis enables accurate detection of *H. pylori* in gastric fluid samples, establishing a novel noninvasive test for *H. pylori* (Tang et al., 2024). Additionally, SERS is resilient against interference from moisture, a common challenge in biological assays, and facilitates real-time detection, which is critical for timely clinical interventions. These attributes render SERS highly amenable to the rapid analysis of biological specimens, including those from bloodstream infections (Chen et al., 2023). The effectiveness of SERS is largely attributable to the significant enhancement of the Raman signal, a process typically achieved through the use of noble metal nanoparticles (NPs) or nanostructures, such as silver (Ag) and gold (Au). In the present study, we harnessed the power of negatively charged nanosilver as an enhancement substrate to amplify the Raman signals across the spectrum of pathogens responsible for bloodstream infections. This approach enabled the successful identification of common pathogens associated with bloodstream infections and their respective drug resistance profiles.

AgNPs’ superior enhancement makes them a favored choice in SERS for bacterial detection, with notable successes including the identification of various *Staphylococcus* species and fungal pathogens (Rebrošová et al., 2017; Witkowska et al., 2018). Wang X. et al. (2023) demonstrated the detection of *E. coli* using an aptamer-modified Au@macroporous magnetic silica photonic microsphere (MMSPM) 3D SERS-activated substrate. Concurrent efforts focused on monitoring bioactive metabolites secreted by ampicillin-resistant *P. aeruginosa* strains, revealing resistance mediated by the pigment pyoverdine (PYO) (Wang and Vikesland, 2023). To mitigate the impact of silver nanoparticle size and aggregation on Raman spectroscopy signals, we employ a series of strategies, including precise control over synthesis conditions for uniform particle size, utilization of stabilizing agents to prevent aggregation, ultrasonication for temporary dispersion, and centrifugation to remove aggregates. Despite these advances, pure culture-based SERS still has limitations in meeting clinical diagnostic speed requirements, for example, the reliance on pure culture bacteria in SERS spectroscopic characterization does not fully satisfy the immediate diagnostic requirements of clinicians, who are faced with additional time constraints for blood plate and culture procedures. Addressing this, research has shifted towards SERS detection from complex samples. Studies have shown promise in isolating bacteria from blood using reagents like Triton X-100 and in developing rapid antibiotic susceptibility testing methods (Lorenz et al., 2019). However, most SERS applications remain in the lab, with few translating to clinical use. A 2023 study by Tseng et al. introduced a SERS-DL model for identifying bacteria from clinical blood cultures, highlighting the need to overcome spectral interferences from blood components like hemoglobin (Tseng et al., 2023).

Considering the imperative for rapid and accurate bacterial separation from blood, this study meticulously compared differential centrifugation with erythrocyte lysis techniques. The results, illustrated in the dot plot (Figure 4), revealed that differential centrifugation yielded SERS spectra with greater detail, facilitating superior differentiation of bacteria from other microorganisms. The application of a CNN model, bolstered by a five-fold cross-validation of accuracy, further confirmed the superior efficacy of differential centrifugation for bacterial isolation. While both methods face challenges in purifying bacterial samples from blood components, erythrocyte lysis, which primarily targets red blood cell removal, may not sufficiently eliminate other interfering substances that could obscure subtle SERS signals of certain bacteria (Croxatto et al., 2014). On the other hand, differential centrifugation, despite its initial limitations, benefits from a secondary round that effectively addresses the impact of specific blood components, enhancing the clarity of SERS spectra (Arana et al., 2023). This study’s findings are of profound significance, identifying a simplified, cost-effective, and more efficient method for bacterial enrichment in BSIs. This approach not only speeds up pathogen detection and resistance analysis but also promises to advance the time to diagnosis of BSI by 24 to 48 h over traditional methods by eliminating the need to subculture pathogen cultures after reporting positive blood culture bottles. This substantial time savings could markedly improve the prompt and effective management of BSIs, marking a pivotal step forward in clinical diagnostics. Our system utilizes the synergy of SERS and CNNs to achieve a more straightforward and faster identification process compared to FilmArray, Verigene, and ePlex technologies. While these platforms are powerful, our approach requires only a small number of samples and simple pre-treatment for immediate results, potentially shortening the time it takes to adopt appropriate antibiotic therapy in BSIs management.

CNNs have emerged as powerful tools in various AI applications, particularly excelling in end-to-end learning processes. Their ability to automatically extract features from raw data has significantly reduced reliance on manual feature engineering. CNNs have shown remarkable effectiveness when applied to unprocessed spectral data, outperforming traditional machine learning approaches in several studies (Liu J. et al., 2017). This prowess is evident in the classification of pathogenic bacteria through SERS spectral analysis. Tang et al. (2022) highlighted CNNs’ superior predictive capabilities across 15 genera of bacteria using SERS spectra. The consistent and stable prediction accuracy of CNNs further solidified their reputation for classifying a broad spectrum of pathogens. This was reinforced in 2023 when a research team trained multiple machine learning and deep learning algorithms on a dataset of 3,126 SERS spectra from 11 Candida species. The one-dimensional (1-D) CNN analyzed the Candida spectral dataset with an overall accuracy of ≥80%, underscoring its robust generalizability (Fernández-Manteca et al., 2023). In the current study, the CNN model’s performance was benchmarked against RF, SVM, and Adaboost, revealing superior identification accuracies of 98.68 and 95.75% for spectra obtained through differential centrifugation and erythrocyte lysis, respectively. The CNN model was also assessed for its ability to isolate bacteria from positive blood culture bottles. Both differential centrifugation and erythrocyte lysis methods produced SERS spectra with high accuracy (ACC ≥95%), indicating strong classification potential. However, the differential centrifugation method outperformed erythrocyte lysis across five key evaluation metrics: ACC, AUC, precision (Pre), recall, and F1, suggesting its superiority in bacterial isolation from blood culture bottles.

The rise of CRKP is a significant threat to the treatment of BSIs, as it leads to higher mortality rates and challenges in anti-infective therapy. Traditional methods for detecting carbapenem resistance, including phenotypic assays like AST, mCIM, and eCIM, are often time-consuming. Molecular assays targeting carbapenemase genes, such as GeneXperT CarbaR, mNGS, and NG-Test Carba5, while more accurate, require specialized equipment and can be costly, limiting their accessibility in healthcare. In this study, a CNN model was developed and demonstrated exceptional performance in identifying carbapenem resistance in *K. pneumoniae* with 99.85% accuracy and an AUC of 100%, using differential centrifugation to obtain SERS spectra. This CNN model outperformed RF, SVM, and Adaboost in classifying pathogens and detecting drug-resistant bacteria. The success of this CNN model suggests that, when integrated with current detection methods, it could offer a more efficient and accurate approach to identifying pathogen drug resistance in the future.

This study conducted a thorough evaluation of two bacterial isolation methods—erythrocyte lysis and differential centrifugation—from positive blood culture bottles of patients with BSIs. The differential centrifugation method outperformed erythrocyte lysis by more effectively eliminating the confounding effects of blood components, thereby enhancing the acquisition of SERS spectral signals from infected pathogens. This approach significantly streamlines the process for subsequent pathogen identification. By integrating a CNN model with SERS, this study harnessed the model’s robust potential for rapid pathogen identification and drug resistance prediction in clinical BSIs. This method offers a marked advantage over traditional culture-based identification by reducing the detection timeline by at least 24 to 48 h. It also lays the groundwork for future direct pathogen detection from mid-stream clinical samples, promising for the early diagnosis and management of BSIs. Despite these advancements, the study acknowledges limitations, including a modest sample size and a restricted range of pathogens examined, which may not encompass all clinically relevant BSI pathogens. Due to the interference of multiple bacteria on the Raman assay results and the fact that we did not collect enough polymicrobial blood cultures, this part of the blood culture was not evaluated for the time being. Further validation with larger, multi-center clinical trials is necessary to confirm the method’s broad applicability and discriminatory power. Nonetheless, the study’s findings are promising, particularly the CNN model’s demonstrated ability to rapidly identify pathogens in reported positive blood cultures, offering a glimpse into the future of direct pathogen detection from BSI specimens and potentially transforming clinical practices in the field.

## Data availability statement

The original contributions presented in the study are included in the article/supplementary material, further inquiries can be directed to the corresponding authors.

## Author contributions

HK: Writing – original draft, Writing – review & editing. ZW: Methodology, Validation, Writing – original draft. JS: Methodology, Validation, Writing – review & editing. SS: Data curation, Methodology, Validation, Writing – original draft. LC: Data curation, Software, Writing – original draft. YS: Formal analysis, Software, Writing – original draft. XP: Formal analysis, Software, Writing – original draft. CW: Data curation, Formal analysis, Software, Writing – review & editing. PG: Data curation, Formal analysis, Software, Writing – review & editing. HL: Writing – review & editing, Conceptualization.
